# Five- and 18-Year Outcome of Two Cases with Full-Arch Rehabilitations *Ad modum* All-on-4 in the Presence of Challenging Conditions

**DOI:** 10.1055/s-0044-1787961

**Published:** 2024-07-23

**Authors:** Miguel de Araújo Nobre, Diogo Santos, Ana Ferro, Armando Lopes, Carolina Jorge Antunes, Inês Vitor

**Affiliations:** 1 Department of Research, Development and Education, Maló Clinic, Lisbon, Portugal; 2 Department of Oral Surgery, Maló Clinic, Lisbon, Portugal

**Keywords:** dental implants, immediate function, full-arch, All-on-4, infection, dehiscence, cyst

## Abstract

Placing implants in fresh postextraction sites is a borderline rehabilitation procedure. The purpose of this report is to describe the pre-, per-, and postoperative procedures for maintaining long-term stability of two full-arch rehabilitations through the All-on-4 protocol, performed in the presence of challenging conditions. Two patients were referred for full-arch rehabilitation with immediate function, with both patients presenting infection in the jaws: patient 1 with an implant (position #45) inserted in a cystic cavity; patient 2 with one implant (position #24) inserted transsinus after the removal of a cyst on the base of the maxillary sinus and another implant (position #15) inserted with a dehiscence. Both patients received a preoperative dental hygiene appointment, a regenerative surgical protocol, and were enrolled in a postoperative maintenance protocol. After surgery a provisional prosthesis was provided ensuring immediate function, and 6 months after surgery, the final prosthesis was delivered. During the follow-up appointments (final follow-up at 5 and 18 years), the implants were stable, and no infection was observed for both patients. The present case report describes two full-arch rehabilitations in immediate function, supported by dental implants inserted in the presence of challenging conditions that do not represent the norm, rather are highly demanding for the clinical team, warranting caution in the interpretation of the results.

## Introduction

The placement of implants in fresh postextraction sites has called for special attention, 1 2 3 and the possible presence of an infection in an extraction site is often seen as a contraindication for implant placement, especially at immediate function, where implant, abutment, and provisional crown (bridge) are placed. 4 Nevertheless, some studies demonstrate that immediate function implants can be placed successfully into extraction sockets provided careful preoperative care is granted 5 and even at infected sites. 6 7 8 9 10 11 12 13 14 15

Traditionally, before placing dental implants, teeth are removed, and the extraction sockets are left to heal for a period of time. 16 To preserve the alveolar bone level from collapse and reduce treatment time, some clinicians began installing the implant immediately after extraction of teeth presenting endodontic or periodontal lesions. 7 8 11 13 17 18 In several studies, there is support for the hypothesis that implants may be successfully placed in infected sites when provided with appropriate clinical procedures before implant surgery such as meticulous cleaning, socket curettage/debridement, and chlorhexidine rinse. 8 17 19 A technique for surgical debridement to reduce and limit bacterial contamination is required, given bacteria can persist as a contaminant in healed alveolar bone following extraction of teeth with apical pathology. 20 Although controversial, some evidence supports using systemic antibiotics and/or guided bone regeneration to fill the bone-implant gap and/or socket deficiencies. 19 21 To reduce surgical treatments and time between tooth extraction and the placement of the definitive prosthesis, immediate implant placement is a valid and supported technique. 18 When primary stability is achieved, several studies demonstrated similar success rates for implants placed into infected sites compared with implants in noninfected or pristine sites. 21 22 23 24 25 26

The All-on-4 concept (Nobel Biocare AB, Gothenburg, Sweden) constitutes a treatment alternative for full-arch rehabilitations supported by implants in immediate function, allowing good long-term treatment outcomes in both arches. 27 28 In these two case reports, the authors describe the surgical technique, pre- and postoperative procedures for placing immediate function implants in challenging conditions for support of full-arch rehabilitations *ad modum* All-on-4 concept (Nobel Biocare AB).

## Case Reports

The present cases illustrate the per-operative, postoperative, and follow-up workflow for full-arch rehabilitations in two patients demanding immediate function rehabilitations (patient 1: mandibular rehabilitation; patient 2: maxillary rehabilitation) following the All-on-4 concept (Nobel Biocare AB) with the implant sites presenting infection at the surgical phase. This research was performed accordance with the Declaration of Helsinki. Both patients provided written informed consent for participating and for the publication of this case report.

### Patient 1

**Fig. 1:**
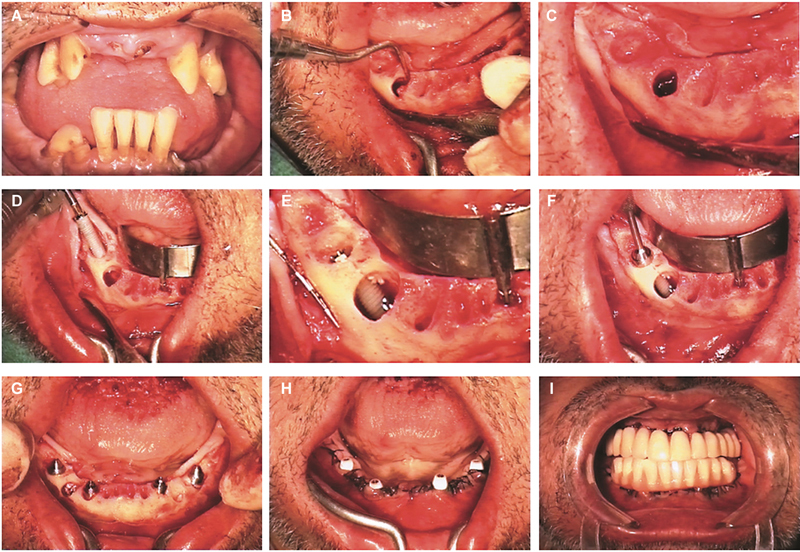
Clinical case illustration of the full-arch rehabilitation ad modum All-on-4 concept in an infected site using per-operative intraoral photographs. ( **A** ) Pretreatment; ( **B** ) view after teeth extraction, bone regularization, and during cyst curettage; ( **C** ) identifying of the mental foramina before the insertion of the posterior distally tilted implant; ( **D** ) immediate postextraction posterior implant insertion with the aid of the edentulous guide; ( **E** ) posterior implant final seating with only coronal and apical bone coverage: Note the cystic cavity of significant dimensions; ( **F** ) connection of the 30 degrees angulated multiunit abutment to the posterior implant; ( **G** ) all implants inserted ad modum All-on-4 concept with two anterior implants placed with axial orientation and two posterior implants distally tilted. Bone remnants from bone regularization were placed inside the cystic cavity; ( **H** ) after suture with the healing caps connected; ( **I** ) the provisional implant-supported acrylic resin prosthesis was connected on the day of surgery achieving immediate function.

A 56-year-old male patient, weighting 100 kg, with hypertension, smoking habits (10 cigarettes per day) was referred for complete rehabilitation of the maxilla and mandible with a fixed prosthetic rehabilitation supported by implants in immediate function. The patient was periodontally compromised, nonbruxer, presenting high masticatory forces and low dental hygiene habits. A full-arch rehabilitation through the All-on-4 concept was proposed. 29 30 The illustration of the clinical situation is presented in Fig. 1 . The natural teeth present in the upper and lower jaws presented infection. These teeth were considered hopeless in terms of periodontal support. One set of teeth (43–44) presented a large infection, with a cystic cavity of 15 mm (mesial-distal) by 10 mm (apical-coronal) of size.

The bone volume was assessed through a cone beam computed tomography scan at the sites where implants were to be inserted, with type 1 bone quality (Lekholm and Zarb). 31 The lack of an alternative for the insertion of an implant on the posterior region of the fourth quadrant to achieve a proper prosthetic support deemed necessary to assess the size of the cystic cavity so to place the implant in that site. The patient received a dental hygiene appointment with explanation of the treatment phases and maintenance procedures at the preoperative phase. The surgical procedure was performed under local anesthesia with mepivacaine chlorhydrate with epinephrine 1:100,000 (Scandinibsa 2%, Inibsa Laboratory, Barcelona, Spain). The patient was sedated with diazepam (Valium 10 mg, Roche, Amadora, Portugal) prior to surgery. Antibiotics (amoxicillin 875 mg + clavulanic acid 125 mg, Labesfal, Campo de Besteiros, Portugal) were given 1 hour prior to surgery and daily for 6 days thereafter. With the objective of controlling the anti-inflammatory response, cortisone medication (prednisone [Meticorten Schering-Plough Farma, Lda, Agualva- Cacém, Portugal], 5 mg) was given daily in a regression mode (15 to 5 mg) from the day of surgery until 4 days postoperatively, and anti-inflammatory medication (ibuprofen, 600 mg, Ratiopharm, Lda, Carnaxide, Portugal) was administered for 4 days postoperatively starting on day 4. Analgesics (clonixine [Clonix, Janssen-Cilag Farmaceutica, Lda, Barcarena, Portugal], 300 mg) were given on the day of surgery and postoperatively for the first 3 days if needed. Antacid medication (omeprazole, 20 mg, Lisboa, Portugal) was given on the day of surgery and daily for 6 days postoperatively.

The patient was informed that the surgical area should be kept cold and under a slight pressure for the first 48 hours after the surgery and was instructed to ingest soft and cold food only during this time period. The insertion of the implants (Brånemark System, Mk IV 4 × 15 mm, Nobel Biocare AB) followed standard procedure for the All-on-4 concept, 30 with underpreparation of the drill sequence to achieve maximum apical anchorage, and a final insertion torque of at least 32 N-cm to accept the implant for immediate function. The two tilted implants were inserted just before the foramina and tilted distally ∼30 degrees, emerging at the second premolar position. The two anterior implants were inserted axially. This arrangement allowed for both optimal implant anchorage and optimal prosthetic support. 29 30 The implant placement was assisted with a surgical guide (Edentulous guide, Nobel Biocare AB), fasten to a 2-mm guide osteotomy in midline of the mandible, with the titanium band shape to follow that occlusal center line of the opposing jaw. The teeth were extracted, and curettage and bone recontouring were performed to keep the infection to a minimum. The implant platform position was aimed to be 0.8 mm above bone level, corresponding to the lower corner of the cylindrical part of the implant flange and bicortical anchorage was established. For the implant in position Federation Dentaire International (FDI) 45, the bone coverage was located only in the coronal and apical thirds. A final insertion torque of >40 N-cm was registered for all implants (50 N-cm for implant in position FDI 45) enabling immediate function. Thirty degrees multiunit angulated abutments (Nobel Biocare AB) were connected to the posterior tilted implants, considering that the right angulation was achieved when the prosthetic screw was at the occlusal aspect of the prosthetic crowns. For the anterior axial implants, straight multiunit abutments (Nobel Biocare AB) were connected. The soft tissues, presenting a thick gingival biotype, were readapted and sutured back into position with 4/0 nonresorbable suture aiming to provide an adequate coverage of keratinized mucosa around the implants. Impressions were taken and the dental laboratory manufactured a provisional high-density baked all-acrylic resin prosthesis with titanium cylinders, connected on the day of surgery, achieving immediate function.

The postoperative maintenance protocol was considered of crucial importance. Inflammation of the wound was attempted to be reduced to a minimum to avoid breakdown of hard and soft tissues, contributing to the installation of an opportunistic infection. Therefore, together with the medication protocol previously described, the patient was instructed to follow a protocol for maintenance of the rehabilitation: For the first 10 days, applying a chlorhexidine gel (Elugel, Pierre Fabre, Lisboa, Portugal) using a postsurgical toothbrush with soft bristles (Elgydium 7/100, Pierre Fabre) and using a mouthrinse (Eludril, Pierre Fabre). From the 10 days until the 4 months postoperatively, the patient was instructed to replace the toothbrush (Elgydium 15/100, Pierre Fabre) while maintaining the remaining products and adding the Superfloss (Oral-B, Lisbon, Portugal). The patient follow-up was carefully performed, with clinical dental hygiene appointments for implant evaluation at 10 days, 2 months, 4 months, and 6 months postsurgically, corresponding to the implants healing phase during the functional osseointegration period.

The final prostheses were delivered 6 months after surgery. These prostheses were full-arch Malo Clinic Acrylic Bridges, composed of acrylic resin crowns (Premium crowns, Mondial Crowns; Heraeus Kulzer GmbH, Hanau, Germany), CAD/CAM-fabricated titanium framework (Nobel Biocare AB) with pink acrylic resin (PallaXpress Ultra, Heraeus Kulzer GmbH) that replicated the missing gingival tissues.

**Fig. 2:**
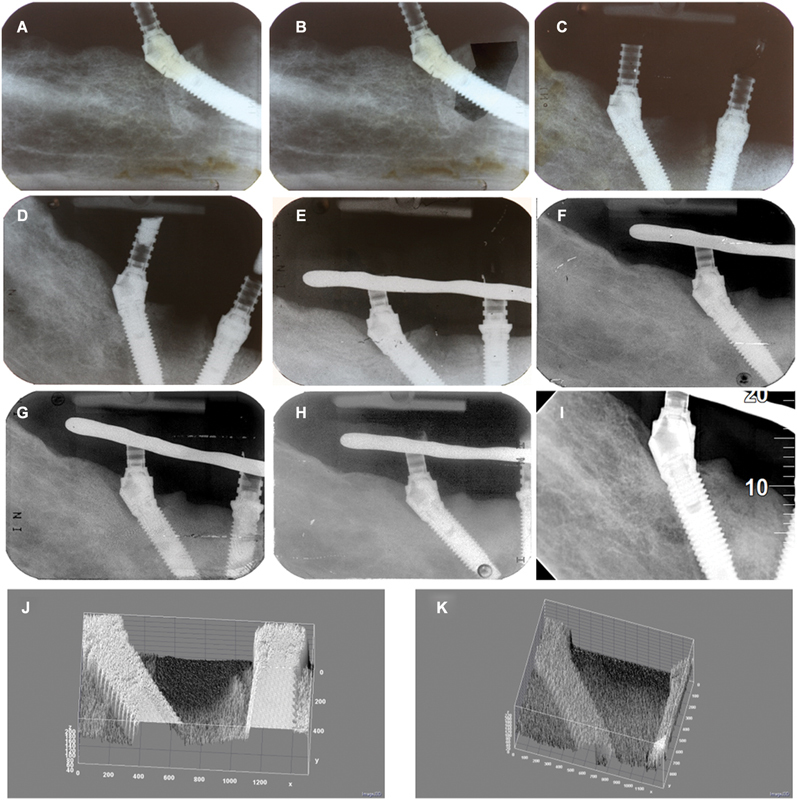
Radiographic control of the case between baseline and 18-year post-surgery between implants #42 and #45. ( **A** ) Baseline on the day of surgery; ( **B** ) baseline on the day of surgery with enhanced contrast; ( **C** ) 1-month postsurgery; ( **D** ) 6-month postsurgery; ( **E** ) 1-year postsurgery; ( **F** ) 3-year postsurgery; ( **G** ) 5-year postsurgery; ( **H** ) 8-year postsurgery; ( **I** ) 18-year postsurgery; ( **J** ) surface plot (Image J) illustrating the baseline condition with lack of bone coverage between the implants; ( **K** ) surface plot (Image J) illustrating the 18 years evaluation with full bone coverage between the implants.

Immediately after the connection of the definitive prostheses, a control appointment was performed and thereafter every 6 months. In these appointments, special attention was given to diagnosis with evaluation of implant stability (clinical mobility evaluated manually), and peri-implant clinical indexes (suppuration, plaque index, bleeding index, and probing pocket depth), together with regular occlusion controls. This meant removing the prosthesis at least once per year. The evolution of the treatment at 1, 5, 10, and 18 years is detailed in Table 1 and was characterized by mild plaque and inflammation levels (Mombelli et al), 32 healthy probing pocket depths and stable marginal bone loss. It is possible to observe the bone healing evolution in the periapical radiographs taken at surgery, and up to 18 years postsurgically ( Fig. 2 ). The surface plot enables to visualize the remarkable recovery of implant 45 ( Fig. 2j, k ). After 18 years of follow-up, the implant in position 45 was stable, with no signs of peri-implant infection and stable marginal bone levels. The remaining implants remained stable throughout the follow-up period up to 18 years.

**Table 1:** Clinical and radiographical evaluation parameters for patients 1 and 2 during the follow-up of the study

Time	Modified plaque index (mPLI) a	Modified bleeding index (mBI) a	Pocket depths	Marginal bone loss in mm (mesial/distal)
Patient 1				
1 y	1	0	≤ 3 mm	0.3 mm/0 mm
5 y	0	1	≤ 3 mm	0.3 mm/0 mm
10 y	1	1	≤ 3 mm	0.3 mm/0 mm
18 y	1	1	≤ 3 mm	0.3 mm/0 mm
Patient 2				
1 y	1	1	≤ 3 mm	FDI 14: 0.9 mm/0 mm FDI 24: 0.3 mm/0 mm
5 y	0	0	≤ 3 mm	FDI 14: 0.9 mm/0.3 mm FDI 24: 0.3 mm/0 mm

Notes: mPLI: 0—no detection of plaque; 1—plaque only recognized by running a probe across the smooth marginal surface of the implant; 2—plaque can be seen by the naked eye; 3—abundance of soft matter. mBI: 0—no bleeding when passing the probe along the gingival margin adjacent to the implant; 1—isolated bleeding spot visible; 2—bleeding forms a confluent red line on margin; 3—heavy or profuse bleeding.

a According to Mombelli et al (1987). 32

### Patient 2

**Fig. 3:**
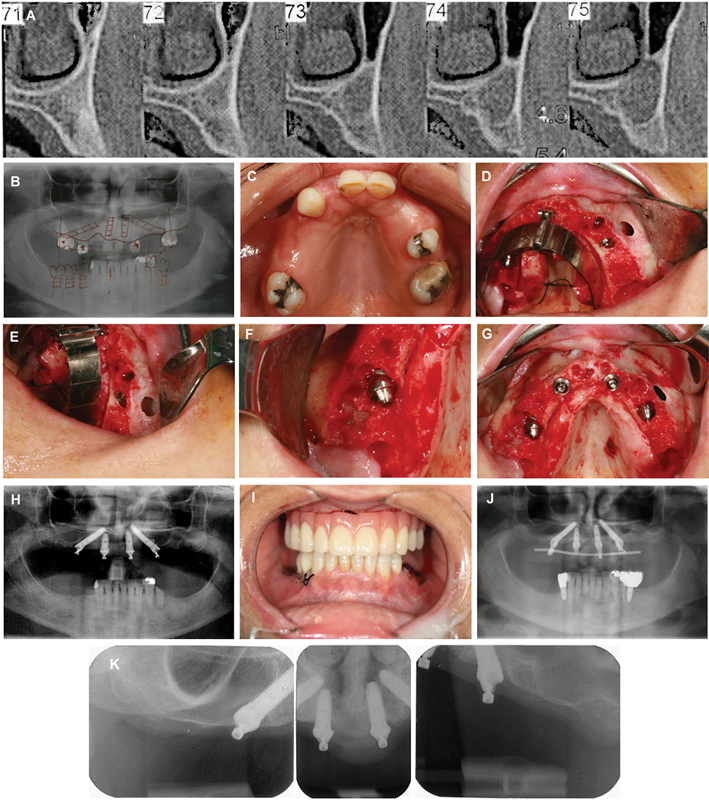
Clinical case illustration of the maxillary full-arch rehabilitation ad modum All-on-4 concept in an infected site: ( **A** ) Pretreatment computed tomography scan slice evidencing the cyst; ( **B** ) pretreatment orthopantomography; ( **C** ) intraoral occlusal view of the maxilla; ( **D** ) per-operative intraoral photograph after tooth extraction and insertion of implant #24; ( **E** ) per-operative intraoral lateral view of the cystic cavity after insertion of the implant #24; ( **F** ) per-operative intraoral view after implant insertion and abutment connection in position #15 (note the dehiscence); ( **G** ) per-operative intraoral occlusal view after implant insertion and abutment connection; ( **H** ) postoperative orthopantomography; ( **I** ) postoperative photograph after the connection of the provisional prosthesis achieving immediate function; ( **J** ) orthopantomography after 5 years of follow-up; ( **K** ) periapical radiographs at 5 years of follow-up.

A 50-year-old female healthy patient, weighting 65 kg, was referred to the clinic seeking full-arch maxillary rehabilitation in immediate function. The natural teeth present in the upper jaw presented infection and were considered hopeless in terms of periodontal support, particularly the premolar on position #15. The patient was nonbruxer and presented low masticatory forces and low dental hygiene habits. The region of the canine (position #23) presented a cyst of 12.9 × 9.0 mm located on the base of the maxillary sinus. Considering the impossibility of inserting implants in the posterior regions that could provide proper prosthetic support while coping with the patient desire for immediate function, made it necessary to plan the tilted implants for positions #15 and #24. The illustration of the clinical situation is presented in Fig. 3 .

The preoperative assessment, surgical procedure, prosthetic provisional, and the maintenance followed the same protocol as described for patient 1. In brief, teeth were extracted, curettage and bone recontouring were performed to keep the infection to a minimum, and the cyst was removed. The patient presented type 3 bone quality (Lekholm and Zarb) 31 and a thick gingival biotype. The insertion of the four implants (NobelSpeedy, Nobel Biocare AB; posterior tilted implants: 4 × 15 mm; anterior axial implants: 4 × 10 mm) followed standard procedure for the All-on-4 concept 29 : The sequence was performed to achieve maximum apical anchorage, and a final insertion torque of at least 32 N-cm to accept the implants for immediate function. The two posterior implants were inserted just before anterior wall of the maxillary sinus and tilted distally ∼30 degrees, emerging at the second premolar (implant #15) and first premolar (implant #24) positions. The two anterior implants were inserted in the regions of the central incisors. This arrangement allowed for both optimal implant anchorage and stable prosthetic support. 29 30 The implant platform position was established with a 2-mm dehiscence in implant #15; flush to bone level in implant #24 (with its coronal portion inserted transsinus); and bicortical anchorage was established in both situations. A final insertion torque >40 N-cm was achieved for all implants enabling immediate function (40–50 N-cm for implant in position FDI 24). A provisional high-density baked acrylic resin prosthesis with titanium cylinders was connected on the day of surgery achieving immediate function. The patient received a reinforced high-density baked acrylic resin prosthesis (PallaXpress Ultra, Heraeus Kulzer GmbH) and acrylic crowns (Mondial crowns; Premium crowns, Heraeus Kulzer GmbH) after 6 months.

Control appointments were performed at 10 days, 2 months, 4 months, and 6 months postsurgically and thereafter every 6 months evaluating implant stability, oral hygiene, peri-implant clinical indexes and occlusion control as described for patient 1. The treatment evolution at 1 and 5 years of follow-up was characterized by an improvement plaque and inflammation levels from mild to absent (Mombelli et al) 32 while maintaining stable levels of marginal bone levels and healthy probing pocket depths ( Table 1 ; Fig. 2 ).

## Discussion

The current clinical cases illustrated the insertion of immediate function implants in the presence of challenging conditions (postextraction sockets and infected sites with the presence of cysts). The treatment of the infection was performed on the same surgical step as the implant insertion through curettage, bone recontouring (that potentially reduced/eliminated the locus of infection), and a medication protocol, to keep the infection to a minimum; choosing anodically oxidized surface implants, a surgical protocol enabling high primary anchorage and a strict maintenance protocol. These procedures provided stability for the implants to overcome the functional osseointegration period (first 6 months of function) and throughout the follow-up until achieving the long term. This outcome was previously reported for shorter follow-ups in several investigations. 5 6 18 22 Maló et al in a study evaluating the 1- to 5-year outcome of dental implants inserted in active periodontitis sites reported good outcomes for partial and complete edentulous rehabilitations. The authors attributed this outcome to the use of a standardized protocol (extraction of teeth/roots, curettage, and small incision to maximize the blood supply), together with the use of oxidized surface implants that allowed to increase implant survival rates to levels comparable to uncompromised periodontal situations. 6 In a systematic review, Chrcanovic et al 33 concluded that implants could be successfully osseointegrated when placed in postextraction of teeth presenting endodontic and periodontal lesions. The authors highlighted the need to carry out appropriate clinical procedures (meticulous cleaning, socket curettage/debridement, and chlorhexidine 0.12% rinse) before the implant surgical procedure to achieve a successful outcome. 33 A recent meta-analysis corroborated this result, registering no significant difference between immediate and delayed implant function when inserted in fresh extraction sockets. 34

Nevertheless, the results of the present case descriptions should be interpreted with caution as they may not represent the norm for long-term outcome of full-arch rehabilitations in these challenging conditions. A previous long-term study of full-arch rehabilitations supported by implants inserted in the presence of dehiscence and fenestrations registered a lower 10-year cumulative success rate for implants placed in fenestrations (87.6%). 35 Furthermore, especially when combined with smoking habits (a combination that occurred in patient 1 of the present case descriptions) resulted in 25% of patients with at least one implant failure. 35

The control of bacteria is vital for maintaining a stable implant condition from the day of surgery until the long term. Concerning the bacterial persistence in dentoalveolar bone following extraction, although this biofilm is of low virulence, it can be activated upon implant placement leading to acute infection. 20 Nevertheless, there was evidence that debridement reduced the number of persistent bacteria in the previously infected periapex area. 20 In the two reported cases, the per-operative protocol consisted of extracting the tooth, performing socket curettage, bone reduction, and disinfecting the area, with the aim of minimizing infection, providing a platform as stable as possible for implant insertion. Consequently, the postoperative protocol served to maintain the stable condition achieved at surgery focusing on diagnosis and controlling the patients by adapting the recall regimen to their risk profile. A previous study attested that patients with implant-borne removable and fixed restorations require professional recall regimes throughout their lives to provide biological and mechanical maintenance. 36 Another study registered significantly higher implant survival rate, lower prevalence of peri-implantitis and peri-implant mucositis for a supportive treatment group with regular maintenance compared with a nonsupportive treatment group. 37 Since both patients in our report received a strict maintenance protocol with regular recall appointments, it was expected that infection, implant failure, and peri-implant pathology would be less likely, contributing to a higher probability of implant survival. The patients' self-care plays an important role in reducing the probability for the occurrence of a peri-implant infection. The high level of plaque control of both patients may have significantly impacted marginal bone level stability. This finds parallel in the literature where a previous study reported a relation between unsatisfactory oral hygiene levels and increased bone loss in complete edentulous patients. 38 Furthermore, considering that the best treatment strategy for peri-implant pathology is prevention, 39 adequate pre- and postoperative care is paramount to achieve and maintain a stable condition. Other factor that may have potentially contributed to the good outcome of both cases was the presence of a thick gingival biotype. Two systematic reviews assessing the influence of soft tissue thickness on marginal bone levels revealed significant differences, with increased marginal bone loss (0.50–1.26 mm) 40 41 for implants with absence of keratinized mucosa irrespective of the implant positioning (crest level or above crest level). 41

Finally, a factor to account for the maintenance of long-term success is an accurate diagnosis through the assessment of clinical and radiological parameters in the professional clinical appointments, allowing to assess the patient's oral health risk. 36 42 As in the present cases, this risk was estimated by measuring infection levels, residual periodontal pockets, loss of periodontal support, tooth loss, systemic conditions, and behavioral factors, such as smoking. 42 Considering this risk, patients with implant rehabilitation require long-term professional care to guarantee both mechanical and biological maintenance. This recall should be adjusted to each patient, since different materials, surgical techniques, and patients at-home care require different needs and maintenances. 36

No inference nor validation can be drawn from these case reports, as they provide only an illustration, do not represent the norm, and therefore caution is advised given the high clinical demanding conditions. As a living tissue, it makes clinical sense to provide bone with function, enabling the stability for implant-supported restorations and increasing the probability of successful outcomes. Proper designed studies with adequate sample size are necessary to improve the treatment outcomes in rehabilitations where there is the need to place implants in compromised areas, paying special attention to surgical and maintenance protocols.

## Conclusion

These two case reports illustrate the technique for placing immediate function implants in postextraction sockets and infected sites with special focus on the pre-, per-, and postoperative procedures to achieve and maintain stability. They do not provide any inference nor validation, and long-term studies with increased sample size are necessary to accurately assess this alternative.
